# The Biomechanics of the Track and Field Sprint Start: A Narrative Review

**DOI:** 10.1007/s40279-019-01138-1

**Published:** 2019-06-17

**Authors:** Neil Edward Bezodis, Steffen Willwacher, Aki Ilkka Tapio Salo

**Affiliations:** 1 Applied Sports, Technology, Exercise and Medicine Research Centre, Swansea University, Bay Campus, Crymlyn Burrows, SA1 8EN UK; 2 Institute of Biomechanics and Orthopaedics, German Sport University Cologne, Am Sportpark Müngersdorf 6, 50933 Cologne, Germany; 3 Department for Health, University of Bath, Bath, BA2 7AY UK; 4 CAMERA, Centre for the Analysis of Motion, Entertainment Research and Applications, University of Bath, Bath, BA2 7AY UK; 5 KIHU, Research Institute for Olympic Sports, Rautpohjankatu 6, 40700 Jyväskylä, Finland

## Abstract

The start from blocks is a fundamental component of all track and field sprint events (≤ 400 m). This narrative review focusses on biomechanical aspects of the block phase and the subsequent first flight and stance phases. We discuss specific features of technique and how they may be important for a high level of performance during the start. The need to appropriately quantify performance is discussed first; external power has recently become more frequently adopted because it provides a single measure that appropriately accounts for the requirement to increase horizontal velocity as much as possible in as little time as possible. In the “set” position, a relatively wide range of body configurations are adopted by sprinters irrespective of their ability level, and between-sprinter differences in these general positions do not appear to be directly associated with block phase performance. Greater average force production during the push against the blocks, especially from the rear leg and particularly the hip, appears to be important for performance. Immediately after exiting the blocks, shorter first flight durations and longer first stance durations (allowing more time to generate propulsive force) are found in sprinters of a higher performance level. During the first stance phase, the ankle and knee both appear to play an important role in energy generation, and higher levels of performance may be associated with a stiffer ankle joint and the ability to extend the knee throughout stance. However, the role of the sprinter’s body configuration at touchdown remains unclear, and the roles of strength and anatomy in these associations between technique and performance also remain largely unexplored. Other aspects such as the sex, age and performance level of the studied sprinters, as well as issues with measurement and comparisons with athletes with amputations, are also briefly considered.

## Key Points

**Table:** 

Although there appears to be no universal optimum body configuration in the “set” position, medium block spacings, which facilitate hip extension and a substantial rear leg contribution, should be encouraged.
Shorter block exit flight times and longer first stance contact times are evident in higher performing sprinters.
During the first stance phase, a “stiff” ankle joint and energy generation by the knee extensors appear to be important features of performance.

## Introduction

Sprinting is a pure athletic endeavor of global appeal, with the 100 m race considered one of the blue-ribbon events at the Olympic Games. The 100 m Olympic final is broadcast worldwide to a potential audience of billions, and athletes from 83 different nations competed in the 100 m event (across both sexes) at the 2016 Olympic Games. At the start of any sprint event, sprinters commence from starting blocks, against which they must produce considerable acceleration. World-class 100 m sprinters can achieve around one-third of their maximum velocity in around only 5% of total race time by the instant they leave the blocks, and sprint start performance is strongly correlated with overall 100 m time (e.g., Baumann [1], Mero [2], Bezodis et al. [3]). Although a previous comprehensive review of sprint start biomechanics was published in this journal by Harland and Steele [4] in 1997, a wide range of descriptive, experimental and theoretical studies have since been undertaken. Many of these have used advanced technologies and methods to identify and understand several new important features of technique for sprint start performance. There is therefore a clear need to review the current understanding of the biomechanics of the track and field sprint start to provide current recommendations for both researchers and practitioners.

### Delimitations of the Review

**Fig. 1:**
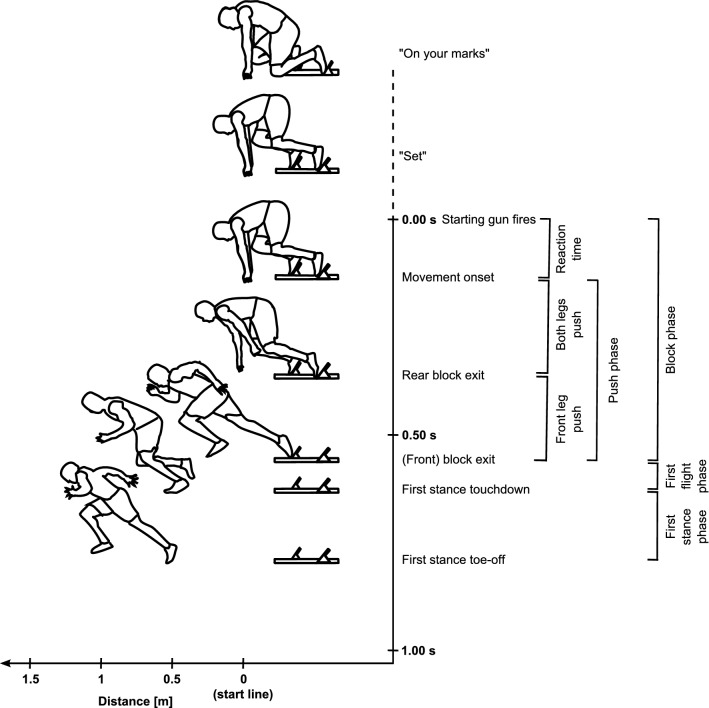
A schematic representation and definition of the events and associated phases during the sprint start, described using the terminology applied consistently throughout this review. The positions of the images are scaled for both horizontal displacement (horizontally) and time (vertically). Event timings are based on data from world-class male athletes during competition [27, 122] aside from the relative timing of rear block exit [3]

The “sprint start” is seldom clearly defined. Studies have typically focused on the block phase and/or one or more of the subsequent steps. In our review, the “start” is only used as a general term. We focus specifically on the block phase and the first flight and stance phases (Fig. 1). Literature from subsequent steps is discussed to provide additional context where relevant. We refer to participants’ ability levels based on reported personal best (PB) 100 m times to avoid the subjectivity associated with inconsistent ability level descriptors (e.g., elite or well-trained).

The articles discussed in this review were initially sourced using a combination of “topic” field search terms (sprint* AND (start* OR accelera* OR block*)) in Web of Science (the final search took place on 21 November 2018). All full papers in peer-reviewed journals were initially retained before one author screened all titles and abstracts to reject clearly irrelevant articles. The remainder were then briefly reviewed by all three authors to identify relevant primary research articles (including the use of starting blocks and spiked shoes) for inclusion in the current review. Given the narrative nature of this review, searches through the reference lists of these articles and manual searches through the authors’ own personal reference manager databases were also undertaken to identify any further potentially relevant papers that had not been retrieved through the above search. All potentially relevant articles were then included in a database and were read in full by one author, who then discussed specific aspects of them with the other authors to ensure a consensus was reached regarding their inclusion, where relevant. After creating the first draft of the review, other papers were then sought that related to specific aspects of the review where further evidence was required (e.g., additional context from subsequent steps or phases of the sprint, strength factors in sprinting, etc.).

### Sprint Start Performance

Total time taken is clearly the default, and appropriate, performance measure during an entire sprint. However, objectively defining successful performance during a discrete section such as the start is less straightforward. For example, does reaching a specific short distance (e.g., 5 m) earlier, or reaching this distance slightly later but with a greater instantaneous velocity, represent superior performance? This issue explains why many different performance measures have been used (Table 1) and why some experimental studies have reported apparently conflicting conclusions when multiple performance measures are considered [5–7].

**Table 1:** Studies that have primarily focused on either technical or physical aspects of the “set” position, block phase or first stance of a maximal effort sprint commencing from blocks and that have included a dependent measure of performance. The specific performance measure(s) adopted in each study are identified, along with an overview of the study design and the studied participants

Study details			Participants			Variable(s) used as dependent measures of performance			
Study^a^	Primary focus of study	Design	Sex	Number	Ability level^b,c^	Time to specific distance^c^ or event	Velocity at specific distance or event	Acceleration over specific phase or at given instant	Power over specific phase
Dickinson [13]	“Set” position	Intervention (within-group)	M	26	Trained sprinters	Block exit 2.29 m			
Henry [6]	“Set” position	Intervention (within-group)	M	18	5.75–6.75 s for 45.72 m	Block exit 4.57 m 9.14 m 45.72 m	Block exit		
Sigerseth and Grinaker [18]	“Set” position	Intervention (within-group)	M	28	Physical education students	9.14 m 18.29 m 27.43 m 36.58 m 45.72 m			
Stock [19]	“Set” position	Intervention (within-group)	M	26	High school athletes	18.29 m 45.72 m			
Menely and Rosemier [114]	“Set” position	Intervention (within-group)	M	30	Physical education students	9.14 m 27.43 m			
Baumann [1]	Block phase	Between-group comparison	M	30	10.2–10.6 s (*n* = 12) 10.9–11.4 s (*n* = 8) 11.6–12.4 s (*n* = 10)	Block exit 5 m 20 m	Block exit	Push phase average and maximum	
Gagnon [115]	“Set” position	Intervention (within-group)	F	6	12.1–13.6 s (*n* = 4) 10.8–11.7 s for 80 m (*n* = 2)	Block exit 50 m	Block exit	Push phase average	
Mero et al. [26]	Block phase and first two stance phases	Between-group comparison	M	25	10.8 ± 0.3 s (*n* = 8) 10.8 ± 0.4 s (*n* = 9) 11.5 ± 0.3 s (*n* = 8)	Block exit	Block exit 2.5 m		Push phase average
Hafez et al. [116]	Block phase	Multiple-single-subject comparison	M	4	10.9–11.7 s		Block exit		
Vagenas and Hoshizaki [53]	“Set” position	Intervention (within-group)	M	15	Skilled sprinters	Block exit 5 m 10 m 20 m	Block exit		
Mero [2]	Block phase and first stance	Cross-sectional analysis	M	8	10.79 ± 0.21 s	Block exit 10 m	Block exit End of first stance		
Mero and Komi [25]	Block phase and first stance	Cross-sectional analysis	M	8	10.76 ± 0.19 s (Gp 1) 10.82 ± 0.23 s (Gp 2)	Block exit	Block exit End of first stance		
Guissard et al. [22]	“Set” position	Intervention (within-group)	M and F	14 and 3	10.4–11.9 s (all)	Block exit	Block exit	Push phase average	
Schot and Knutzen [15]	“Set” position	Intervention (within-group)	M and F	6 and 6	University intercollegiate track team		Block exit End of first stance 2 m		
Mendoza and Schöllhorn [7]	“Set” position	Intervention (within-group and multiple-single-subject)	M	8	10.4–10.8 s	10 m	Block exit		Push phase average
Čoh et al. [29]	Block phase and first two steps	Cross-sectional analysis and between-sex comparison	M and F	13 and 11	10.73 ± 0.2 s (M) 11.97 ± 2.6 s (F)	5 m 10 m 20 m 30 m	Block exit End of first and second stance		
Reis and Fazenda [117]	“Set” position	Cross-sectional analysis	M	15	Sprinters	20 m 60 m			
Salo and Bezodis [11]	Block phase (vs. standing)	Intervention (within-group)	M and F	4 and 2	10.98 ± 0.40 s (M) 12.55 ± 0.35 s (F)	25 m 50 m	10 m 25 m		
Fortier et al. [57]	Block phase and first two steps	Between-group comparison and intervention (within-group)	M and F	16 and 4	10.46 ± 0.11 s (Gp 1) 11.07 ± 0.30 s (Gp 2) 7.60 ± 0.46 s (60 m; Gp 3)	4 m	Block exit Average first stance	Push phase and first step peak	
Gutiérrez-Dávila et al. [56]	Block phase	Intervention (within-group)	M	19	11.09 ± 0.30 s		Block exit		
Mero et al. [23]	“Set” position	Intervention (within-group)	M	9	10.86 ± 0.34 s	20 m	Block exit		
Bradshaw et al. [45]	“Set” position, block phase and first two steps	Group-based description	M	10	10.87 ± 0.36 s	Block exit 10 m			
Maulder et al. [102]	Block phase and first three steps	Intervention (within-group)	M	10	10.87 ± 0.36 s	Block exit 10 m	Block exit	Push phase average	
Čoh et al. [52]	Block phase and first two steps	Single-subject analysis	F	1	13.19 s (100 mH)	Block exit	Block exit End of first and second stance		
Bezodis et al. [5]	Block phase	Cross-sectional analysis	M	12	11.30 ± 0.42 s	10 m 20 m 30 m	Block exit 10 m 20 m 30 m	Push phase average	Push phase average
Bračič et al. [104]	Block phase	Cross-sectional analysis	M	12	10.82 ± 0.25 s		Block exit		
Slawinski et al. [24]	Block phase and first two steps	Between-group comparison	M	12	10.27 ± 0.14 s (Gp 1) 11.31 ± 0.28 s (Gp 2)	5 m 10 m	Block exit	Push phase average and peak First stance average and peak	
Aerenhouts et al. [94]	Block phase (and first five steps)	Between-group comparison	M and F	39 and 28	10.81 ± 0.40 s (M s) 11.29 ± 0.29 s (M j) 11.85 ± 0.24 s (F s) 12.54 ± 0.26 s (F j)	Block exit	Block exit 5 m 10 m 15 m 20 m	Push phase average	
Charalambous et al. [70]	First stance phase	Single-subject analysis	M	1	13.48 s (110 mH)	5 m	Change during first stance End of first stance 5 m		
Slawinski et al. [16]	Block phase and first step	Intervention (within-group)	M and F	6 and 3	10.58 ± 0.27 s (M) 11.61 ± 0.42 s (F)	Block exit End of first stance 5 m 10 m	Block exit End of first stance		
Ille et al. [37]	Block phase	Intervention (within-group) and between-group comparison	M	16	Skilled sprinters, football players and basketball players	Block exit 10 m			
Okkonen and Häkkinen [118]	Block phase	Cross-sectional analysis	M	9	11.35 ± 0.29 s	10 m	Block exit		
Debaere et al. [30]	Block phase and first two steps	Group-based description	M and F	11 and 10	10.62 ± 0.18 s (M) 11.89 ± 0.30 s (F)		Block exit End of first and second stance		
Bezodis et al. [71]	First stance phase	Multiple-single-subject comparison	M and F	2 and 1	10.14–10.28 s (M) 12.72 s (100 mH; F)				First stance phase average
Milanese et al. [44]	Block phase and first two stance phases	Intervention (within-group)	M and F	6 and 5	12.0 ± 0.1 s (M) 13.1 ± 0.9 s (F)	Block exit	Block exit Start of first and second stance		
Otsuka et al. [46]	Block phase and first two steps	Between-group comparison	M	29	10.87 ± 0.41 s (Gp 1) 11.31 ± 0.42 s (Gp 2) Nontrained (Gp 3)	2 m		Push phase average	
Taboga et al. [54]	Block phase	Intervention (within-group) and between-group comparison	M and F	13 and 3	12.49 ± 1.11 s (M and F, able-bodied) 13.17 ± 1.31 s (M and F, with amputation)	Block exit	Block exit	Push phase average	Push phase average
Debaere et al. [86]	First and second stance phases	Group-based description based on simulation model	M and F	2 and 5	11.10–11.77 s (M) 12.05–12.36 s (F)			Maximal acceleration during first stance phase	
Otsuka et al. [20]	Block phase	Intervention (within-group)	M	14	10.99 ± 0.40 s	Block exit 2 m	Block exit	Push phase average	Push phase average
Bezodis et al. [3]	Block phase	Cross-sectional analysis	M	16	10.95 ± 0.51 s				Push phase average
Bezodis et al. [68]	First stance phase	Theoretical intervention based on simulation model	M	1	10.28 s				First stance phase average
Chen et al. [119]	Block phase	Intervention (within-group)	M	7	10.94 ± 0.20 s		Block exit End of first stance (vertical component only)		
Schrödter et al. [21]	Block phase	Cross-sectional analysis and between-group comparison	M and F	54 and 30	10.98 ± 0.58 s (M) 12.12 ± 0.68 s (F)				Push phase average
Willwacher et al. [82]	Block phase	Cross-sectional analysis and between-group comparison	M and F	103 and 51	9.58–14.00 s (all able-bodied) 12.24 ± 0.33 s (all athletes with amputation)	Block exit	Block exit		Push phase average
Ciacci et al. [27]	Block phase and first two steps	Between-group comparison	M and F	10 and 10	10.03 ± 0.14 s (M 1) 10.74 ± 0.21 s (M 2) 11.10 ± 0.17 s (F 1) 11.95 ± 0.24 s (F 2)		Block exit		
Čoh et al. [120]	Block phase and first two steps	Between-group comparison	M	12	10.66 ± 0.18 s (Gp 1) 11.00 ± 0.06 s (Gp 2)	Block exit 4 m	Block exit	Push phase average First stance phase average	
Debaere et al. [95]	Block phase through until start of second touchdown	Between-group comparison	M and F	21 and 22	10.65 ± 0.07 s (M s) 11.21 ± 0.11 s (M j) 11.56 ± 0.08 s (M a) 11.87 ± 0.14 s (F s) 12.42 ± 0.25 s (F j) 12.86 ± 0.30 s (F a)		End of first stance		
Janowski et al. [98]	Block phase	Multiple-single-subject comparison	M	2	10.33 and 10.39 s	20 m			
Piechota et al. [61]	Block phase	Between-group comparison	M	54	Expert sprinters and physical education students	Block exit 5 m 10 m 30 m			
Aeles et al. [73]	First stance phase	Between-group comparison and cross-sectional analysis	M and F	18 and 19	10.67 ± 0.14 s (Ms) 11.47 ± 0.34 s (Mj) 12.12 ± 0.41 s (Fs) 12.75 ± 0.36 s (F j)		Δ*v* during first stance		
Brazil et al. [59]	Block phase	Cross-sectional analysis	M	17	10.67 ± 0.32 s				Push phase average
Strutzenberger et al. [113]	First and second stance phases	Between-group comparison	M	22	10.10–11.20 s (able-bodied) 11.70–12.70 s (with amputation)	5 m 10 m	First step average		
Wild et al. [12]	First three steps	Cross-sectional analysis (and between-group comparison)	M	18	10.60 ± 0.40 s				First stance phase average
Bezodis et al. [50]	Block phase	Cross-sectional analysis	M	23	11.37 ± 0.37 s				Push phase average
Bezodis et al. [72]	First stance phase	Group-based description and between-group comparison	M	17	10.66 ± 0.32 s (able-bodied) <12.50 s (T36)				First stance phase average
Sandamas et al. [63]	Block phase and first stance	Intervention (within-group) and cross-sectional analysis	M and F	8 and 2	11.03 ± 0.36 s (M) 11.6 ± 0.45 s (F)	Block exit	Start of first stance End of first stance		Push phase average First stance phase average

*Gp* group,  *mH* meter hurdles, *M* male, *F* female, *s* senior, *j* junior (< 18 years), *a* adolescent (< 16 years), *T36* paralympic athlete in T36 category

^a^Studies are included in chronological order. Although some studies (e.g., Wild et al. [12], Debaere et al. [86]) also included acceleration or power measures in subsequent steps, these measures were only included in the respective studies because they also focused on technical aspects during these subsequent steps, and thus these later measures are deemed irrelevant for the purposes of this table. Kistler [14] was not included in this table because, although it investigated a technical “set” position intervention, the dependent measure was a representation of mean pressure exerted against the blocks. Cavagna et al. [121] was not included in this table as it focused on the energetics over 30 m rather than specifically on the “set” position, block phase or first stance, but it was the first study to consider average external power as an outcome measure

^b^Ability levels of participants are reported as 100 m personal best times (to the precision reported in the original study) where possible. All other descriptors are verbatim from the methods section of the cited study

^c^All distances have been converted into metric units

The most common measure of sprint start performance has been center of mass (CM) velocity at block exit (i.e., block velocity; Table 1). Block velocity is determined by push phase impulse and can therefore be increased by either greater force or greater time spent producing force. The ability to produce force is not consistent throughout the duration of (and range of motion covered during) the push against the blocks. Therefore, there comes a point when attempting to achieve further increases in block velocity by simply pushing for longer against the blocks may not be beneficial for overall sprint performance (i.e., the least possible time to cover a given distance). In an attempt to overcome this limitation, average external power production has been proposed as an objective performance measure during any part of the start [5]. Average external power, which is typically calculated based on horizontal motion and normalized to participant characteristics, provides a single measure that accounts for the change in velocity and the time taken to achieve this change (i.e., the rate of change in kinetic energy) [5]. This performance measure has since been adopted in numerous sprint start studies (Table 1) and during early and mid-acceleration [8, 9].

## The “Set” Position

Sprinters can choose the location and inclination of two foot plates in a block start [10]. Although three-point or standing starts are of interest for relay events and athletes in other sports, performance during standing starts differs from that out of blocks [11], as do the techniques adopted by sprinters and team sports athletes from their respective starts [12]. Our review therefore focuses on studies of sprint-trained athletes starting from blocks.

### Foot Plate Spacings

Increasing the antero-posterior distance between the foot plates leads to increased push phase duration and total impulse and therefore greater block exit velocities [6, 13–15]. This is likely due to greater rear leg forces, which lead to greater rear leg segmental kinetic energies [16]. However, as block velocity is a potentially biased performance measure, whether these effects actually represent an improvement in push phase performance is less clear. Despite eliciting greater block velocities, elongated starts (mean inter-block spacing = 0.548 m) lead to longer times to 5 and 10 m compared with bunched (0.215 m) and medium (0.368 m) starts [16]. Bunched starts reduce the extension capability of both hips and the rear knee, whereas during elongated starts the longer push duration cannot necessarily be used favorably for generating force [17]. Medium block spacings therefore appear to provide the most favorable basis for push phase performance because they allow sprinters to generate relatively large forces without spending overly long doing so [16, 18, 19]. However, definitive block spacing recommendations remain challenging because of different performance measures and spacings used between studies (bunched is typically < 0.3 m, medium between 0.3 and 0.5 m, and elongated > 0.5 m [4]), and because little consideration has been given to sprinter anthropometrics.

Wider medio-lateral foot plate spacings (0.45 m) affect hip joint kinematics (particularly non-sagittal) compared with typically used block widths (0.25 m), but do not affect block power [20]. Although the International Association of Athletics Federations (IAAF) does not specify limits to block width [10], given that sprinters are required to use starting blocks provided by the organizers in competition, that no manufacturer currently makes medio-laterally adjustable blocks, and that there appears to be no performance benefit of adjusting the medio-lateral spacing [20], there is limited need for further exploration in this area.

### Foot Plate Inclination

There is no effect of habitual foot plate inclination on block power when analysed cross-sectionally across a wide range of sprinters [21]. Front block inclination is also not related to any external force parameters, but a steeper rear foot plate is associated with a greater mean rear block horizontal force between sprinters [21]. However, when analysed within sprinters (10.4–11.9 s), experimental reductions in front block inclination (from 70 to 30°, relative to the track) acutely increase block velocity (from 2.37 to 2.94 m/s) without significantly affecting push phase duration (mean increase = 0.004 s [22]). Furthermore, concomitant reductions in both foot plates’ inclinations (from 65 to 40°) also lead to acute increases in block velocity (3.30 vs. 3.39 m/s) within sprinters (10.86 ± 0.34 s [23]), but this is accompanied by a slightly greater (0.010 s), albeit non-significant, increase in push phase duration. Foot plate inclination affects plantar flexor muscle-tendon mechanics during the block phase [23], and the range of dorsiflexion and mean dorsiflexor stretch velocities achieved are both positively correlated with block power (*r* = 0.38–0.70 [21]). This potential conflict between cross-sectional [21] and within-sprinter [22, 23] evidence could also arise from differences in foot plate surface lengths between studies. The identification of individual-specific foot plate inclinations that facilitate initial dorsiflexion may therefore be important, but future research should also consider the effects of different commercially available foot plate surface lengths.

### Joint Angular Kinematics

A sprinter’s block settings combine with their anthropometrics to affect “set” position body configuration. Although a general position is typically now evident, with the hips above the shoulders and the shoulders ahead of the start line [24, 25], “set” position joint angles from groups of sprinters across different ability levels have led to the identification of positions adopted by subgroups of faster sprinters. These include more flexed hips (mean = 41° and 80° vs. 52° and 89° for the front and rear legs, respectively, between fastest and slowest groups [26]), more extended rear knees (136° vs. 117° [24]) or more flexed front knees (99° vs. 91° [27]). However, it must be considered that considerable variation is typically evident in “set” position kinematics between sprinters, even within relatively homogeneous groups across studies spanning a range of ability levels [2, 3, 25–30], and only weak or nonsignificant correlations exist between lower body joint angles in the “set” position and block power [3]. Differences in “set” position kinematics between groups determined by 100 m times may therefore be an effect of other factors that are important for overall sprint performance and consequently influence a sprinter’s choice of “set” position. It is likely that no single, universally optimum combination of lower body joint kinematics exists when in the “set” position [3, 27], and other contributing factors (e.g., anthropometry, strength [26]) should be explored.

## The Push Phase

Reaction times vary greatly between and within sprinters [25, 31, 32] and do not differ based on ability level [24]. Other factors, such as disqualification rule changes [33], holding time [34, 35], start signal intensity [36], and the sprinter’s focus of attention [37], can also affect reaction times. Whilst excitation of lower limb muscles occurs prior to the first visible movement or force production against the blocks ([25, 38] see Sect. 3.3), and a sprinter’s ability to react is undeniably important, a more detailed discussion of the factors related to the processes that occur between the start signal and movement initiation is beyond the scope of our review; this section therefore focuses on motion during the push phase. Having reacted, the aim of the push phase is to maximize horizontal velocity in as little time as possible. Sufficient vertical impulse must also be produced to overcome gravity and initiate a gradual rise [39], and > 85% of this vertical block exit velocity is produced during the phase where both legs push [30]. After rear block exit, the front leg must also assist vertical motion, but its primary role therefore appears to be forwards propulsion.

### Kinematic Considerations

During the push phase, both ankles typically initially dorsiflex, whereas both knees and hips solely extend [3, 21, 30, 40]. The front leg exhibits a proximal-to-distal peak angular velocity sequencing [3, 40, 41] consistent with that typically observed during extension tasks [42, 43]. However, in the rear leg, the knee reaches peak angular velocity before the hip then ankle [3, 40, 41]. This may be because the rear knee starts from a relatively extended angle (e.g., 114–121° [27]) and thus has limited opportunity to extend, and could relate to the aforementioned vertical velocity generation during the rear leg push. Experimental manipulations have shown that rear knee angles of 90° in the “set” position led to higher block velocities in 12.0 s (male) and 13.1 s (female) sprinters than more extended (both 115° and 135°) rear knee angles due to a greater rear block push duration without any change in the overall push phase duration [44]. However, the observed effect may also have been due to compensatory adjustments at the other rear leg joints as block spacings were fixed across all conditions.

Peak angular velocity magnitudes are variable both within [45] and between [3] sprinters, even within a relatively homogeneous group (10.30 ± 0.14 s [41]). Peak angular velocities at both hips and rear hip range of extension are positively associated with block power (all *r* = 0.49 [3]). Elongated starts are associated with increased peak hip angular velocities [17] and, although a single, universal ideal “set” position may not exist (Sect. 2.3), more elongated block starts may therefore be worth considering for sprinters with limited hip extension. The front hip also demonstrates abduction and external rotation in excess of 100°/s during the final 25% of the push phase [41]. Whilst whole-body transverse plane motion has been found not to differ between groups of 10.87 and 11.31 s sprinters during the push phase or first two steps [46], three-dimensional movements have been described at the joints of both the lower and the upper body [41], and further research is needed to better understand the importance of these non-sagittal joint kinematics.

Upper body push phase kinematics have been the focus of considerably fewer studies. The movements at both shoulder joints are three-dimensional in nature, and the peak resultant angular velocities at the shoulder and elbow joints are comparable to those at both knees during the push phase [41], although upper body angular velocities are considerably more variable (between-sprinter) than lower body angular velocities [41]. These complex upper-limb joint kinematics combine to raise the hands from the ground but, in relation to the torso, each arm’s motion primarily opposes the other from movement onset onwards, and thus their combined direct contribution to forwards acceleration is minimal [47]. It has been proposed that the arms primarily counterbalance lower body rotations but also that vertical arm motion may facilitate leg drive and thus contribute indirectly to forwards acceleration [47]. Although there have been detailed descriptions of the arms’ actions [41], there exists no evidence to relate differences in arm action to sprint start performance levels, and future research in this area may be necessary given the emphasis often placed on it by coaches [48].

### Kinetic Considerations

#### External Kinetics

It has long been known that sprinters with faster PB times [1] and those with higher velocities after 2.5 m [26] generate larger relative horizontal block impulses than their slower counterparts. These impulses are typically achieved despite the same or shorter push phase durations, i.e., they are due to increased average horizontal force production. Subsequent research has identified greater peak and average forces [49, 50] and higher rates of force development [24] as potential explanations. The forces under the hands have also been recorded in some studies [46, 50], but their primary role appears to be one of support [46]. The front leg contributes 66–76% of the total horizontal impulse [51, 52] due to 1.9–2.4 times longer block contact than the rear leg [3, 51, 52]. Group mean block velocities are therefore significantly greater with the stronger leg in the front block (3.37 vs. 3.12 m/s when in the rear block [53]). However, familiarization effects must be considered because acute switches between legs are typically “uncomfortable” or “awkward” [54], and reaction time [55] and total push duration [54, 55] effects must also be considered.

Although the front leg produces greater impulse, larger forces can be achieved against the rear block [51], and rear block force magnitudes are the most predictive external kinetic feature of block power [49, 50]. This includes higher forces throughout the entire rear leg push as well as greater “pre-tension” against the rear block in the “set” position [50], although acute experimental increases to the force against the blocks when in the “set” position do not lead to increases in block velocity [56]. A longer rear leg push (as a percentage of the total push phase) is also positively associated (*r* = 0.53 [3]) with greater block power [3, 50] and evident in sprinters with faster PBs [57]. Maximizing the rear leg impulse contribution therefore appears to be an important strategy, provided it does not elongate the total push phase duration. Another important kinetic feature is the front block direction of force application [46, 49], supporting the aforementioned importance of the front leg for forwards propulsion [30]. However, direction of force application has not been identified as important in all push phase studies [50], possibly due to different study designs or data analysis techniques. Future research should explore this further given the known importance of direction of force application during subsequent acceleration [58].

#### Joint Kinetics

The lower limb joint kinetics underpin the previously discussed joint kinematics, and combinations of average ankle, knee and hip joint moment and power magnitudes during the push phase have been found to explain up to 55% of the variance in block power across 17 sprinters with a mean PB of 10.67 s [59]. Ankle plantar flexion resultant joint moments (RJMs) are dominant in each leg throughout its respective push [40]. There is a small phase of energy absorption followed by energy generation at both ankles, and the aforementioned foot plate inclination effects therefore likely relate to a stretch-shortening cycle mechanism during the early push phase [23]. In the rear leg, there is a negligible knee RJM, but a rear hip extensor RJM is dominant throughout the majority of the push and generates energy [40]. In the front leg, knee RJM calculations have been affected by center of pressure estimation differences [60], but the knee RJM is likely extensor dominant until just prior to block exit, thus generating extensor energy [40, 60]. The front hip is extensor dominant from movement onset before becoming flexor dominant at about 85–90% of the push phase, thus absorbing energy just prior to block exit [40]. Each hip contributes > 60% of the total positive joint work done by the respective leg [40], which reinforces the kinematic evidence regarding the importance of the hips during the push phase, and this likely helps to contribute to the progressive increases in the kinetic energy of the head and trunk segments as the push phase progresses towards block exit [41]. The upper limbs’ translational kinetic energy progressively increases for the majority of the push phase such that the arms possess around 22% of the total body kinetic energy before decreasing during the late part of the pushing phase, whereas the kinetic energy of the lower limbs and trunk continue to increase until block exit [41]. Although it has been suggested that the total kinetic energy of the body could be increased if all segments reached their maximum at the same time [41], this may not be possible because of the sequencing required to transfer energy most effectively between segments [42, 43].

### Muscular Considerations

Whilst muscle excitation can vary considerably between individuals [25], it typically commences prior to horizontal force production against the blocks [25, 38], and the earlier onset of muscle excitation relative to the onset of force production has been positively correlated with maximal horizontal block force and block velocity magnitudes [25]. The rear leg gluteus maximus is typically the first muscle excited during the block phase [25, 52], followed by the rear leg semitendinosus [61] and biceps femoris, and then the quadriceps and calf muscles [25, 51]. The rear leg quadriceps are typically only excited during the early part of the rear leg push; excitation ceases prior to rear block exit to keep this foot clear of the track during the subsequent rear leg swing [51, 52], which may explain the sequencing of peak angular velocities in the rear leg. Whilst the vastii muscles are relatively highly excited during the rear leg push, rectus femoris excitation is less evident [61], which could be due to the importance of rear hip extension during this phase. Towards rear block exit, only the biceps femoris and calf muscles remain excited [51], which is consistent with knee extension being arrested but hip extension and ankle plantarflexion continuing.

In the front leg, the vastii muscles are typically excited soon after the initial gluteus maximus and biceps femoris activation and remain excited almost until block exit [51, 52]. In contrast to the vastii muscles, the rectus femoris muscle only becomes excited during the late push phase [51], where it also helps to arrest hip extension and facilitate the transfer of energy distally down the leg. The front leg soleus is excited considerably earlier than the gastrocnemius muscle [51], which may be due to knee flexion in the “set” position shortening the biarticular gastrocnemius [21, 51]. Whilst the available muscle excitation information is largely descriptive in nature, it provides useful context for determining the specificity of training exercises to the push phase. There is also scope for simulation-based research to explore hypothetical questions regarding the strength and sequencing of these muscle actions.

## The First Flight and Stance

After exiting the blocks, the first stance phase contains the greatest velocity increase during any stance within a maximal sprint [8]. Importantly, achieving high levels of block power is not associated with any potentially detrimental features of technique at first stance touchdown [3], and thus striving to improve push phase performance does not appear to inhibit subsequent technique.

### Kinematic Considerations

#### Spatiotemporal Variables

In both male and female Diamond League competitors (mean PBs = 10.03 and 11.10 s, respectively), the first flight phase lasts just 0.045 ± 0.025 s, and block exit step lengths during this flight are 1.14 m (males) and 1.07 m (females) [27]. These step lengths are greater than those (0.97 and 0.95 m) of groups of 10.74 s (male mean PB) and 11.95 s (female) sprinters analysed using the same methods [27]. Medio-lateral step widths of over 0.3 m (group mean) also occur during this block exit step [46, 62], and restricting block exit step width can reduce horizontal propulsive impulse production (by 0.05 m/s) during the first stance phase [63].

Step frequencies of around 4 Hz are typically exhibited immediately post block exit. When analysed within a single cohort of sprinters across an entire sprint, step frequencies from the first flight and stance are already up to 90–95% of their respective values during maximum velocity [39, 64]. These relatively consistent step frequencies across a sprint reflect the fact that flight times progressively increase and contact times progressively decrease as the acceleration phase progresses [8, 39, 64]. Mean first stance contact times for Diamond League sprinters are 0.210 s (males) and 0.225 s (females), which are greater than those of their lower-level comparators (0.176 and 0.166 s [27]). Combined with their longer block exit step lengths, the CM of higher-ability sprinters is therefore typically further ahead at first stance toe-off than that of lower-level sprinters [24, 27].

Although long contact times are not desirable at maximum velocity, shorter block exit flight times and longer first stance contact times would increase the time during which propulsive force can be generated in this period of high acceleration and reduce the time spent in flight where force cannot be generated. Shorter flight times and longer contact times are also observed in higher-level sprinters in the step immediately after first stance toe-off [27], and this strategy may continue until mid-acceleration where rates of reduction in contact time become associated with performance [65]. However, caution must be applied since simply spending longer in stance to produce the same average force may not be beneficial due to the *least possible time* nature of sprint performance. As faster trials within session and within individual are associated with shorter contact times from the first step onwards [66], the longer contact times of higher-level sprinters are likely more related to longer-term physical adaptations, which facilitate this technical strategy. Coaches must therefore be cognizant of the trade-off between contact time and increases in velocity (i.e., net horizontal impulse) when exploring this.

#### Touchdown Kinematics

At first touchdown, higher performing sprinters typically land with their CM further along the track [24]. The foot is behind the CM at first touchdown (i.e., a negative touchdown distance [3, 9, 26]), and moves progressively forwards relative to the CM at touchdown as a sprint progresses (e.g., by 0.09 m from touchdown one to two, and a further 0.09 m from touchdown two to three [26]). Irrespective of which point on the foot is measured, the CM is behind the stance foot from the third touchdown onwards [9, 26]. Whilst touchdown distance has been related to braking impulse magnitude during the early part of stance in the mid-acceleration phase (16 m) in athletic males [67], the link between touchdown kinematics and ground reaction force features during early acceleration remains poorly understood. This may be because a curvilinear relationship between touchdown distance and stance phase power likely exists [68]. This is due to an inability to produce sufficient magnitude of resultant force with the foot further behind the CM and an inability to direct this force in the required horizontal direction with the foot less far behind the CM [68, 69].

#### Joint Angular Kinematics

Proximal-to-distal sequencing is evident in peak stance leg hip, knee, ankle and metatarsal-phalangeal (MTP) angular velocities during the first stance phase [40, 70–72]. The stance leg MTP joint initially dorsiflexes during the first 10–15% of stance but is then relatively stationary until around 60–65% of stance, after which there is further dorsiflexion followed by a rapid plantar flexion (up to 500°/s), which peaks around toe-off [71, 72].

After leaving the rear block, the ankle joint dorsiflexes throughout the majority of its swing phase, but plantar flexion starts just before touchdown [30]. After touchdown, the ankle dorsiflexes for the first ~ 40% of stance, then continually plantar flexes towards and beyond toe-off [30, 40, 70–73]. Reducing the range of ankle dorsiflexion during early stance has been theoretically demonstrated to increase first stance power [68]; this would require greater plantar flexor RJMs [68] and thus a “stiffer” ankle (see Sect. 4.2.2).

Knee extension of the leg placed in the rear block starts just after midway between rear block exit and first touchdown [30], and thus this stance leg knee extends from the very onset of touchdown [30, 40, 70–73]. This is different from later phases of acceleration [39] and maximum velocity [74], where there is an initial phase of knee flexion during stance. The step in which stance knee flexion first occurs (third to sixth) is closely related to a first transition in the rise of CM height following block exit [39], and thus this solely extension action of the knee during early stance may play a role in the rise of the CM during early acceleration. The stance leg knee continues to extend throughout the majority of stance towards peak extension angles of around 160–170° [30, 70–73], but not to full extension, likely due to both geometrical and anatomical constraints [75]. The transition to knee flexion typically starts within the final 10% of stance [40, 70–72], but this is not consistently the case, with some sprinters still extending their knee at toe-off [30, 71].

Having flexed from soon after rear block exit, the stance leg hip starts to extend slightly before touchdown [30] and continues to extend throughout stance [30, 40, 70–73]. For some sprinters, the hip starts to flex just prior to toe-off [40, 71, 72], although this is not always the case [30, 70, 71, 73]. There is also around 15–20° of hip abduction during stance as well as some internal rotation [30]. Considerable lumbar extension occurs during block exit, and, although it continues during each of the first two stance phases, it is largely negated by lumbar flexion during flight [30, 76]. The gross trunk angle increases observed throughout the acceleration phase [9, 39] therefore appear to be primarily due to a gradually less anteriorly tilted pelvis across the first two steps [30, 76].

### Kinetic Considerations

#### External Kinetics

Whilst the initial braking phase is often short (around 8–13% of total stance time [2, 71, 72]) and peak braking forces can be relatively low (e.g., < 0.17 bodyweight [71]), there is no evidence to suggest that no phase of braking exists during the first stance phase in sprinters of any level. Braking force magnitude has been suggested to be a function of touchdown distance and foot touchdown velocity [77, 78]. Whilst these factors may explain some variation in braking force magnitudes [67], they do not appear to be the sole causes of braking, since braking forces are still observed even when touchdown distances are large and negative and the foot is moving slightly backwards relative to the ground at touchdown [71]. Further research is therefore needed to identify other contributors to braking.

The propulsive phase can contain peak horizontal forces of around 1.3 bodyweight [71], and net propulsive impulses associated with increases in horizontal velocity of between 1.1 and 1.4 m/s are produced during the first stance [2, 71, 72]. Larger propulsive horizontal forces are produced by sprinters of higher performance levels throughout the entire acceleration phase [79], and during early acceleration the production of greater propulsive forces during mid-late stance is particularly important [8]. Larger propulsive forces during early acceleration have also been confirmed as a desirable feature within individual sprinters [66]. Horizontal propulsive forces clearly play an important role in early acceleration performance, but caution must still be applied to ensure that sufficient vertical impulse is produced to overcome the effect of gravity and to continue the gradual rise into upright running [68].

#### Joint Kinetics

MTP RJMs are plantar flexor dominant throughout the first stance [71, 72], consistent with observations during mid-acceleration [80, 81]. Although the modelling of the MTP joint can affect the magnitude of the plantar flexor RJMs and joint work [81], the MTP joint is fairly stationary during the first half of stance before then dorsiflexing, and it is thus a net energy absorber from around mid-stance before generating a small amount of energy as it plantar flexes just prior to toe-off [71, 72]. The MTP RJM is due not only to the musculature crossing the joint but also to passive biological components and external factors such as shoe stiffness. Shoe stiffness has been shown to affect acceleration performance from a standing start [82] and MTP and ankle RJMs during drop jumps [83], but further work is needed to better understand their direct effects on sprint start technique and performance.

An ankle plantar flexor RJM acts throughout the first stance [30, 40, 70–73]. There is therefore an initial phase of energy absorption prior to energy generation, but the ankle can generate up to four times more energy than it absorbs during the first stance [71, 72]. By the 16 m mark, ankle energy absorption is roughly equal to energy generation [84], and in the maximum velocity phase the ankle is a net energy absorber [74], although caution must be applied to direct comparisons between studies because different foot models can affect ankle joint power magnitudes [85]. Induced acceleration analysis has revealed the ankle to be the greatest contributor to CM propulsion during first stance, with plantar flexor action propelling and lifting the athlete throughout stance due to the negative touchdown distance [86]. Greater ankle stiffness during dorsiflexion has also been associated (*r* = 0.74) with higher horizontal CM velocity at toe-off [70], and reduced dorsiflexion has been theoretically demonstrated to increase stance phase power production [68]. The ankle joint therefore appears to play an important role in early acceleration performance. Future work is required to better understand how technical and/or physical training can be implemented to alter the function of the ankle and ultimately enhance sprint acceleration performance.

As the knee joint extends from before first stance touchdown, extensor power could theoretically be generated at the knee joint throughout stance. However, only some sprinters produce knee extensor RJMs at touchdown [30, 40, 70, 71, 73]. Reduced horizontal toe velocities at touchdown may assist the generation of knee extensor RJMs at touchdown [71]. This could increase knee energy generation and ultimately external power production given that the knee is an important energy generator during the first stance phase [30, 70–72]. The positive energy contribution from the knee (relative to the amount produced by the hip and ankle) reduces considerably in the second stance phase [30], and thus knee joint energy generation may be less important in subsequent stance phases. This may be due to one or more potentially related reasons, such as the inability of the knee to extend from touchdown, or the changing touchdown distance and thus greater influence of geometrical constraints [75].

The hip RJM is initially extensor dominant, with peak hip extensor power occurring near touchdown but not consistently before or after it [30, 40, 70–72], although this could be affected by filtering methods [87]. The hip RJM becomes flexor dominant later in stance and, whilst this has most commonly been observed to be at around 65–80% of stance [40, 70–72], standard deviations span between 15 and 80% in other studies [30, 73]. This variation may be due to filtering [87] or hip joint center location [88] differences between studies, although the musculature and physical abilities of trained sprinters due to their specific preparation (e.g., Handsfield et al. [89]) could be an important factor. Physical abilities (see Sect. 5.3) and anatomical factors have often been overlooked in joint kinetic analyses of sprinting. Whilst sprinters have different lower leg anatomy to non-sprinters [90–92], there are no differences between groups of more closely matched (10.27 vs. 10.67 s) sprinters [93], so the role of anatomy in kinetic differences between trained sprinters across ability levels remains unclear.

### Muscular Considerations

After each foot has exited its respective block, the rectus femoris and tibialis anterior muscles are excited in both legs during their respective early swing phases [51] in order to assist hip flexion and ankle dorsiflexion. For the rear block leg, rectus femoris excitation ceases by mid-swing [51, 61] and is replaced by biceps femoris excitation, which may work with the gluteus maximus to assist the reduction of foot touchdown velocity [25, 52, 69]. Several extensor muscles (soleus, gastrocnemius, rectus femoris and the vastii group) are excited just prior to ground contact [51], and whilst these remain highly excited at first stance touchdown, biceps femoris and tibialis anterior excitation cease around touchdown [51]. During stance, muscle-driven induced acceleration analysis has revealed that soleus contributes slightly more to horizontal CM acceleration than the gastrocnemius, but almost twice as much to vertical CM acceleration, potentially due to the biarticular nature of the gastrocnemius [86]. The biarticular gastrocnemius and rectus femoris muscle tendon units, as well as the soleus, stretch then shorten during the first stance phase, whereas the vastii muscles solely shorten [73]. These are ideal conditions for the storage and release of energy, supporting the likely contribution of knee and ankle work to the high levels of first stance power [73] as well as the earlier suggestions of proximal-distal energy transfer. This muscular information again provides a useful reference for exercise selection and development, but there is also scope for further musculoskeletal modelling during the first stance given the less complex nature of modelling ground contact.

## Other Considerations

### Translating Information Between Different Populations

#### Female and Male Sprinters

The majority of the research discussed has focused on male sprinters. Whilst differences in both technique and performance have been reported between males and females [29, 64, 94], these comparisons are typically made between sprinters of the same relative ability within their respective sex. However, when the additive and interactive effects of both sex and absolute ability level are assessed, ability level explains more differences in start kinematics than does sex [27]. The only clear sex differences are that males have a shorter push phase duration, higher block exit velocity and shorter contact times for the first two steps. Caution should therefore be applied to the translation of general biomechanical information between sexes when the absolute performance level has not been accounted for.

#### Junior and Senior Sprinters

Although junior sprinters may lack the muscularity of their senior counterparts, horizontal block force production, block velocity and push phase duration appear not to differ between adult and junior athletes [94]. There is also little difference in push phase joint kinetics between adults, under 18s and under 16s [95]. However, beyond the block phase, adult senior sprinters of both sexes exhibit significantly longer first step lengths and achieve significantly higher velocities at 5 m than juniors [94], possibly because younger sprinters are unable to generate as much knee joint power during stance [95]. Although knowledge regarding the techniques of junior sprinters remains relatively limited, some knowledge gained from the numerous studies of adults may be relevant to junior athletes, particularly during the block phase.

#### Performance Levels

Very few peer-reviewed sprint start studies have analysed truly world-class sprinters (e.g., international finalists) and thus little scientific evidence is available regarding the individual techniques of the fastest athletes on the planet. Whilst caution must be exerted when appraising potentially valuable non-peer-reviewed evidence relating to such individuals, it is also paramount that caution is applied when translating peer-reviewed evidence beyond the ability levels of the studied participants. This is illustrated by the differences in spatiotemporal measures between Diamond League competitors and sprinters closer to the ability levels typically analysed in research (see Sect. 4.1.1 [27]).

### Measurement Issues

The need for greater information on high-level sprinters during competition clearly presents challenges around access, but—where possible—research based on manual video analyses of sprinters in competition should be encouraged [27]. Whilst this will only directly yield kinematic information, it provides context that enables the appropriate comparison and interpretation of kinetic and muscular data collected in more controllable environments. Other technologies have been used, although caution must be applied to data from currently available laser [96] and global positioning system (GPS) [97] devices during early acceleration. Inertial measurement units offer a theoretically promising option for relatively noninvasive data collection during training [98]. However, the error in such data must be critically considered in the context of the smallest meaningful differences for understanding sprint start technique, particularly given the magnitudes of random measurement error previously reported [99, 100].

### Strength Considerations

Our review has largely focused on technical issues with little consideration for the underlying strength characteristics. This is primarily because the biomechanical research we have reviewed has typically focused on reporting kinematic and kinetic features of technique without any additional measures of physical abilities. General and specific measures of physical abilities are positively correlated with push phase (e.g., Mero et al. [26], Debaere et al. [64], Smirniotou et al. [101], Maulder et al. [102, 103], Bračič et al. [104]) and early acceleration (e.g., Mero et al. [26], Sleivert and Taingahue [105], Nagahara et al. [106]) performance levels. However, for further developments to be made in this area and to help inform the application of specific strength training in an attempt to address identified technical flaws, interactions between physical abilities and technique need to be explored [94]. There also exists a wealth of information on the mechanical specificity and acute and longer-term effects of sprint start and acceleration strength training methods, and interested readers are encouraged to read existing reviews such as those by Delecluse [107], Bolger et al. [108], Seitz et al. [109], Cronin et al. [110], Petrakos et al. [111], Rumpf et al. [112].

### Athletes with Amputations

Sprinters with lower-extremity amputation(s) partly lack the ability to generate muscular force. Running-specific prostheses can only store and return but not generate energy, which results in reduced start performance (average reduction in block power = 17.7% [49]) for athletes with amputations. Athletes with unilateral amputations normally position their affected leg in the rear block [49, 54], and although force application from the non-affected front block leg is not necessarily lower, push phase duration is longer, and the forces are more vertically directed [49]. After block exit, athletes with amputations demonstrate reduced step length, step frequency and horizontal force application [113]. These differences, combined with the reduced block phase performance, result in slower 5 m and 10 m times than those of non-amputee sprinters [113].

## Conclusion

This review discusses the available literature that has studied the technical aspects of the block, first flight and first stance phases of a maximal effort sprint from starting blocks. Based on our review, we list several key conclusions and recommendations that we believe are relevant to both researchers and practitioners.

### Summary and Recommendations

- Where possible, average horizontal external power should be used to objectively quantify performance during any discrete part of a sprint start.
- Although a general “set” position is typically evident, no single optimum combination of lower body joint kinematics likely exists for all sprinters. Medium block spacings likely provide the best starting point for maximizing push phase performance because they allow sprinters to generate relatively large forces without spending overly long doing so. Foot plate inclinations that individually facilitate initial dorsiflexion may be important.
- The influence of body configuration, anthropometry and strength, combined with different block settings and “set” positioning, on push phase performance remains poorly understood, although individual block setting manipulations could be informed by the available evidence in attempts to overcome specific technical issues (e.g., increase inter-block spacing for sprinters with low hip angular velocity).
- The extension of both hips appears important for performance during the push phase against the blocks. Maximizing the magnitude and relative duration of rear leg force production may also be an important means through which to increase average horizontal force production during the push phase; greater rear hip extension and a greater early extension from the knee may be important in this.
- Although non-sagittal lower-body motion and arm movements (three-dimensional) have been described in detail during the start, their associations with performance levels are not well understood. Given the frequent emphasis by coaches on arm actions, these in particular should be clarified by future research.
- Shorter block exit flight times and longer first stance contact times are evident in higher performing sprinters; these increase the time during which propulsive force can be generated.
- A “stiffer” ankle joint, which dorsiflexes less during early stance, likely plays an important role in first stance phase performance. More research to understand the effects of ankle-specific technical or physical training on sprint acceleration performance is needed.
- The knee joint is an important energy generator during the first stance phase. This may be because of the body configuration and ability to extend the knee from touchdown in contrast with the later phases of a sprint.
- For all leg joints, the specific role of strength and anatomy in sprint start performance remains unclear and requires further investigation.
